# Supplementary material for: Zero‐Field Anomalous Hall Effect in Bulk Single Crystal Mn3Ir
Source: Adv Sci (Weinh). 2025 Nov 8;12(46):e12559. doi: 10.1002/advs.202512559 (PMC12697786; doi:10.1002/advs.202512559)
Supplement: Supplementary file 1 — Supporting Information [file ADVS-12-e12559-s001.docx]

**Supplementary for “Zero-field Anomalous Hall Effect in Bulk Single Crystal Mn_3_Ir”**

*Xin Gu,* [*Ruoqi Wang*](https://onlinelibrary.wiley.com/authored-by/Wang/Ruoqi)*, Bo Zhao, Haofu Wen, [Kunquan Hong](https://onlinelibrary.wiley.com/authored-by/Hong/Kunquan), [Shijun Yuan](https://onlinelibrary.wiley.com/authored-by/Yuan/Shijun)^1,2^*,* [*Taishi Chen*](https://onlinelibrary.wiley.com/authored-by/Chen/Taishi)*^1,2^*,* [*Jinlan Wang*](https://onlinelibrary.wiley.com/authored-by/Wang/Jinlan)


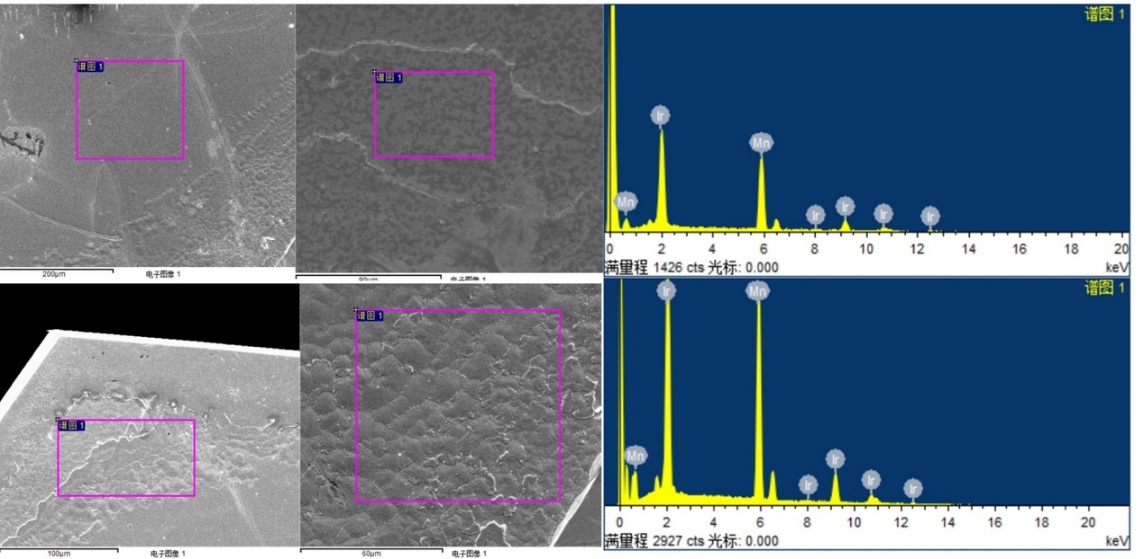


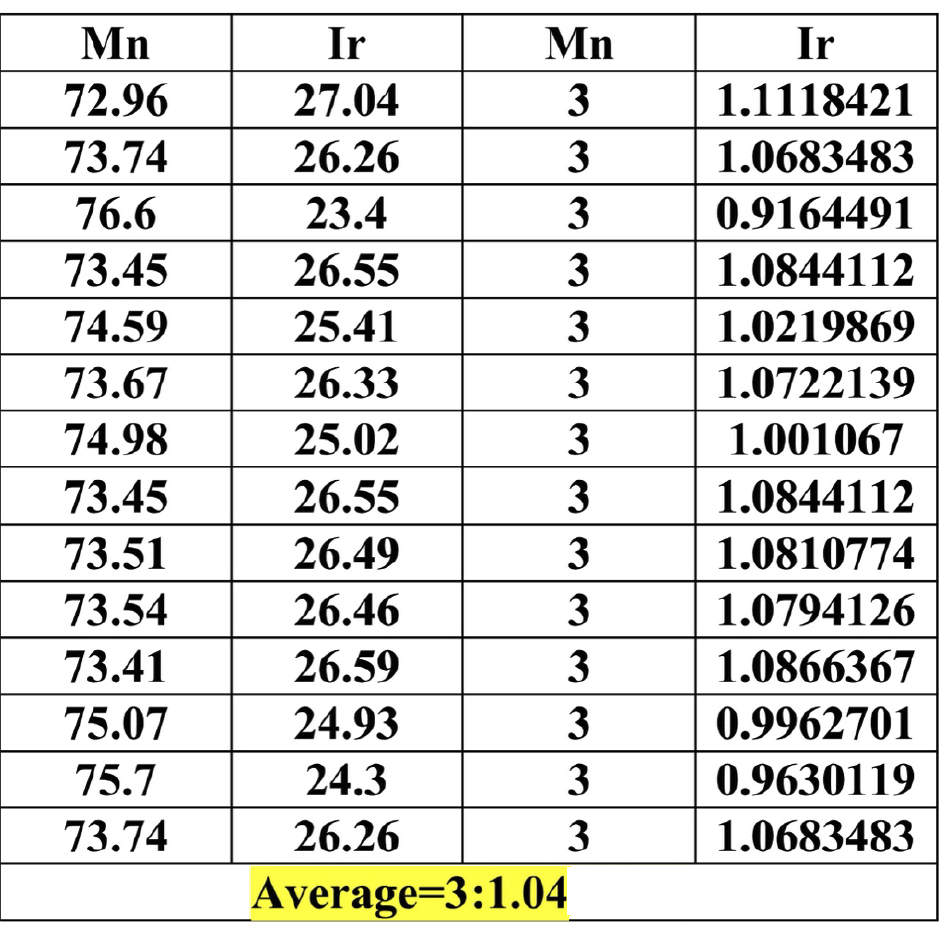


**Figure S1:** SEM/EDS results from the polished Mn_3_Ir bulks, and below is the atomic ratio of Mn and Ir, which yields 3:1.


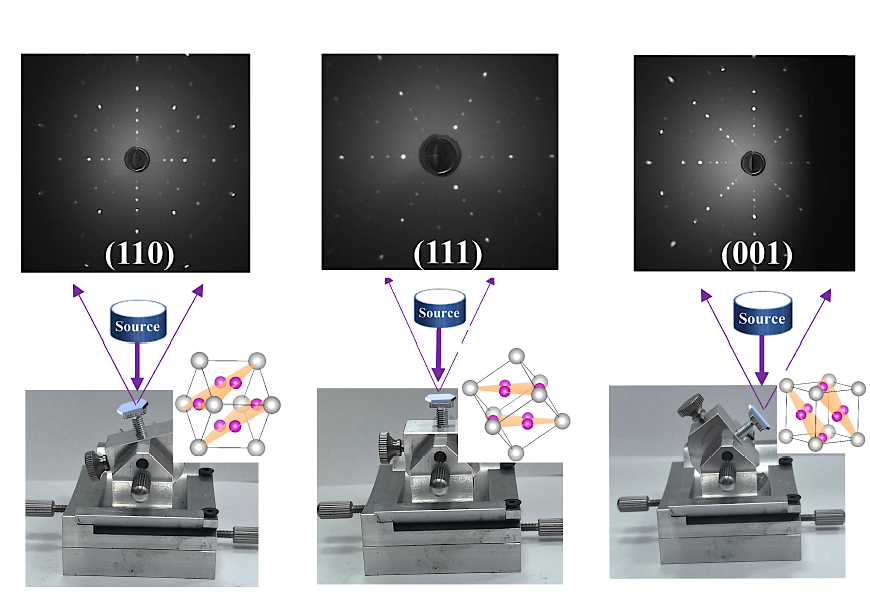


**Figure S2:** Laue patterns and measurement configurations. The orange triangles represent the related position of the Mn_3_Ir samples in the primitive cell.

**
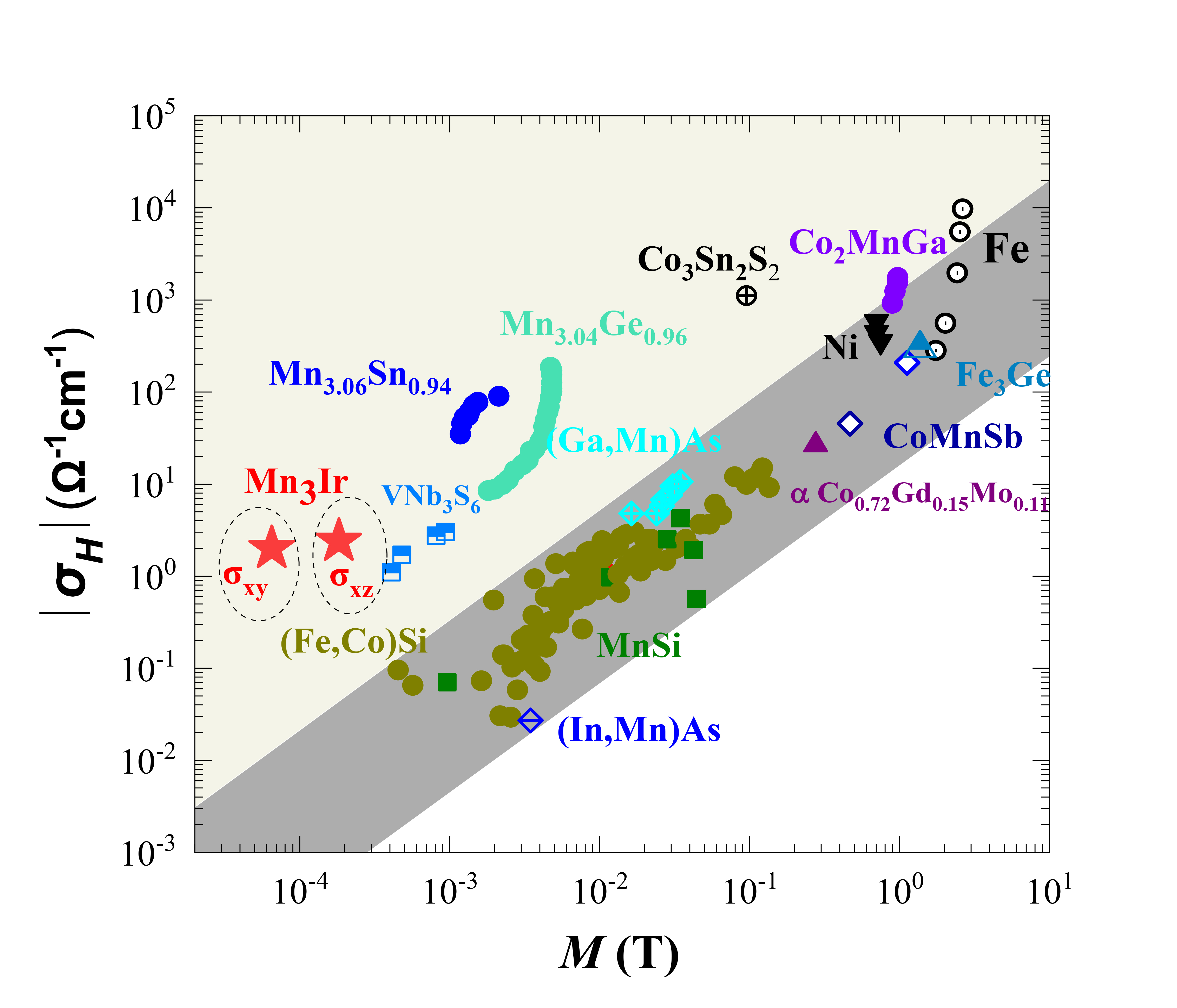
**

**Figure S3**: Scaling plot of anomalous Hall conductivity vs. magnetization. The black-shaded region represents the anomalous Hall effect arising from extrinsic mechanisms, while the light-yellow region corresponds to the intrinsic anomalous Hall effect originating from the Berry curvature.


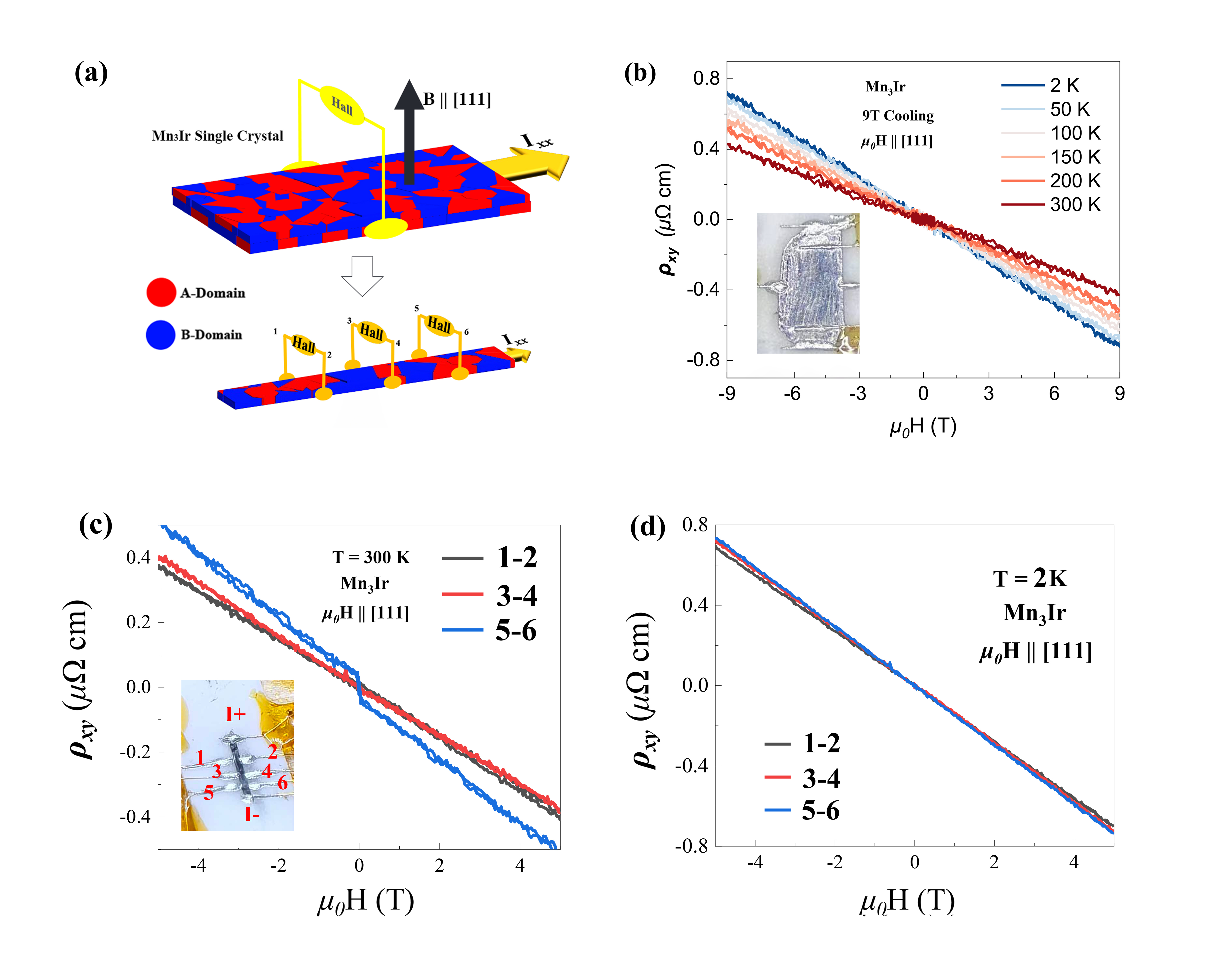


**Figure S4**：Enhanced AHE in miniaturized Mn_3_Ir bulk crystals. a) In large-sized Mn_3_Ir single-crystal bulks, the equal populations of A/B domains cancel the AHE. In contrast, small-sized samples can exhibit an imbalance in the number of A/B domains, leading to an enhanced anomalous Hall effect. b) AHE of large-sized Mn_3_Ir bulk crystals measured at different temperatures: only a very small AHE is observed at 300 K (as shown in the main text). c) and d) AHE measured at different temperatures for a small-sized sample (2.2 mm × 130 *μ*m × 30 *μ*m) with three sets of Hall contacts. Notably, Hall contacts 5–6 show a pronounced enhancement in the AHE.
